# Advanced Merkel cell carcinoma of the lower extremity treated with surgery and isolated pelvic and limb perfusion using Melphalan: A case of unexpected long-term survival

**DOI:** 10.1016/j.ijscr.2019.06.064

**Published:** 2019-07-08

**Authors:** S. Guadagni, A. Chiominto, A.R. Mackay, A.R. Farina, L. Cappabianca, I. Puccica, S. Valiyeva, M. Clementi

**Affiliations:** a Department of Applied Clinical Sciences and Biotechnology, Section General Surgery, University of L’Aquila, 67100, L’Aquila, Italy; b Alma Mater Europaea–European Center, Maribor, Slovenia; c Department of Pathology, San Salvatore Hospital, L’Aquila, Italy; d Department of Applied Clinical Sciences and Biotechnology, Section of Pathology and Clinical Molecular Oncology, University of L’Aquila, 67100, L’Aquila, Italy; e Department of Applied Clinical Sciences and Biotechnology, Section General Surgery, University of L’Aquila, 67100, L’Aquila, Italy

**Keywords:** Merkel cell carcinoma, Merkel cell polyoma virus, In-transit metastases, Melphalan, Isolated pelvic and limb perfusion, Case report

## Abstract

**Introduction:**

Merkel cell carcinoma (MCC) is a rare, neuroendocrine skin tumor, with high frequency of locoregional recurrence, metastases, and poor prognosis. Locoregional MCC recurrence in the extremities can pose considerable treatment challenges. We report a case of long-term survival in a female patient with recurrent MCC of the leg, treated with surgery and locoregional chemotherapy.

**Presentation of case:**

A 73-year-old female with cirrhosis and hepatitis C, developed cutaneous MCC in the left inferior limb. This patient initially received surgical treatment, with subsequent negative sentinel lymph-node biopsy in another center, one-month prior recovery in our department, and arrived with 4 new limb nodules, cranially to the previously treated area, without distant metastases or inguinal lymph node recurrence. This patient was not eligible for immunotherapy due to active hepatitis upon treatment with NS5B inhibitors, or eligible for systemic chemotherapy or radiotherapy due to severe neutropenia and was, therefore, subjected to surgical resection combined with Isolated Pelvic and Limb Perfusion (IPLP) with Melphalan. Histological evaluation confirmed MCC diagnosis and during the following 4 months, she developed further locoregional recurrences with homolateral inguinal lymph node involvement and was subjected to two additional rounds of surgery plus IPLP.

**Discussion:**

All procedures were tolerated, systemic toxicities were temporary and subsequent clinical and radiological follow-up, following the last combined treatment, indicated that this patient was still alive and disease-free, at 56 months.

**Conclusion:**

In this case, surgery combined with locoregional Melphalan chemotherapy was an effective and repeatable treatment for recurrent MMC and resulted in unexpected long-term survival.

## Highlights

• Merkel cell carcinoma is a rare and aggressive skin cancer.
• We report the case of a female patient with recurrent MCC of the leg.
• The patient was not eligible for immunotherapy, systemic chemotherapy and radiotherapy.
• The patient received repeated surgical excisions combined with Melphalan IPLPs with unexpected long-term survival.

## 1 Introduction

Merkel cell carcinoma (MCC) is a rare and aggressive skin cancer. MCC propensity for rapid invasion and dissemination to other regions of the body, underpins its poor prognosis. Surgery followed by radiation therapy is considered the first-line treatment for primary or loco-regional MCC, whereas chemotherapy has always been used to treat advanced forms. However, chemotherapeutic responses, in general, are of short duration with an unclear clinical benefit, considering overall survival rates, underpinning a need for novel therapeutic strategies for the treatment of this tumor type.

Recent advances in understanding the molecular pathways in MCC, combined with development of novel agents that target angiogenic factors, apoptosis inhibitors, poly-ADP ribose polymerase, components of the PI3K/Akt/mTOR pathway, receptors such as the somatostatin receptor, and novel immunotherapy agents, have hailed in a promising new era of targeted therapy. Within this context, the PD-1/PD-L1 immune checkpoint inhibitors Avelumab, Pembrolizumab and Nivolumab have shown promising results in advanced stage MCC, supporting a “Standard of Care” use of these agents for metastatic MCC [1].

Recently, a 53% increased risk of developing non-epithelial, non-melanoma skin cancers, including MCC, has been linked to Hepatitis C infection [2]. In addition, immune suppression has been closely linked to MCC-initiation, illustrated by the higher incidence of MCCs in patients with hematological malignancies, HIV infection, organ transplantation and following immunosuppressive treatment, implicating the fully functional immune system in MCC prevention [3]. MCCs in immune-compromised patients are more aggressive, and in these patients, immunotherapy is not recommended in the presence of local or systemic MCC recurrence [4].

Regional Melphalan-based chemotherapy has been shown to be a safe and effective alternative therapy in MMC patients with local recurrences in the extremities, and associates with occasional durable complete responses [[5], [6], [7]]. Here, we report the unexpected, long-term survival of a MCC patient with local lower limb recurrence, who was subjected to multiple rounds of surgery plus locoregional IPLP Melphalan chemotherapy, in line with the SCARE criteria [8].

## 2 Case presentation

This retrospective case report study has been approved by the investigational review board [Ethics committee of “ASL n.1, Abruzzo, Italy; Chairperson: G. Piccioli; protocol number 10/CE/2018; date of approval: 19 July 2018 (n.1419)].

**Fig. 1:**
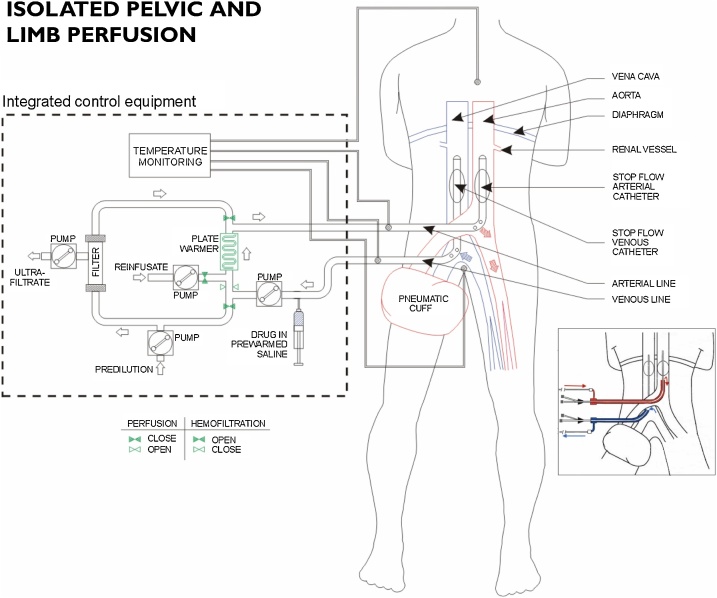
Schema of surgical and percutaneous Isolated Pelvic and Limb Perfusion (IPLP) with chemofiltration.

A 73-years old caucasian women was admitted to our surgical unit with a diagnosis of MCC and suspected cutaneous recurrences in the left lower limb. Two months previously this patient was recovered in another hospital for the surgical removal of a cutaneous lesion, localized in the calcaneal region, confirmed by histology to be an 18 x 7 mm MCC. The patient also underwent sentinel lymph node biopsy, in the same hospital, the results of which were negative. The patient presented with Hepatitis C-related cirrhosis, with portal thrombosis and esophageal varices, treated with Sofosbuvir (400 mg) and Daclastavir (30 mg) and exhibited chronic serious neutropenia due to long-term ionizing radiation exposure, as a result of her profession as a radiographer. Total body CT-scan staging did not reveal systemic disease but 4 subcutaneous sites of recurrence in the leg, cranial to the previously removed primary lesion, were detected by RM. Bilateral ultrasound examination of the inguinal basins did not reveal lymph node involvement (Stage IIIc), and laboratory tests confirmed severe neutropenia (WBC 1.900 cells/mcL), mild thrombocytopenia (PLT 74.000 plateled/mcL) and normal coagulation. The patient was scheduled for wide surgical excision of cutaneous recurrences combined with locoregional Melphalan (30 mg) chemotherapy by way of Isolated Pelvic and limb Perfusion (IPLP), based upon palliative criteria (Fig. 1). The patient was informed of the risks and advantages of the proposed therapeutic approach and provided fully informed consent.

**Fig. 2:**
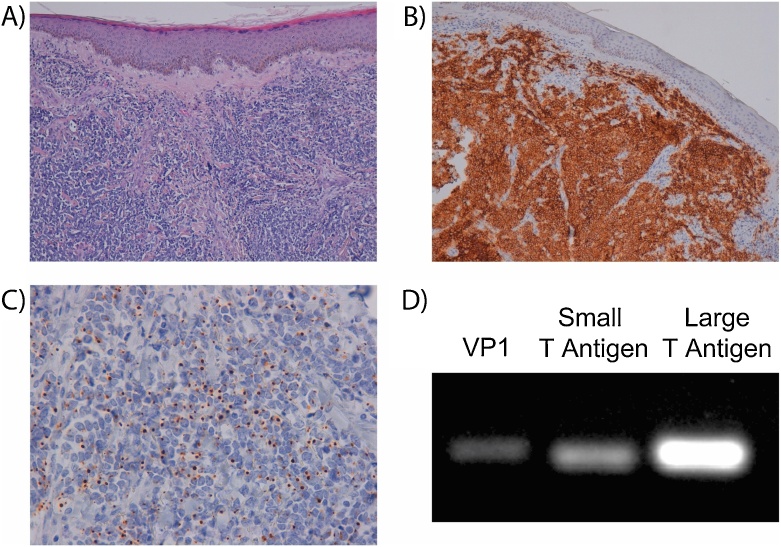
MCC characteristics: A) Haematoxilin and eosin stain 100 × . Monotonous dermal round cell infiltrate and diffuse pattern of infiltration; B) CD56 100 × . Strong and diffuse immunoreactivity; C) Cytokeratin 20 400 × . Dot-like immunoreactivity; D) RT-PCR demonstrating for MCPyV VP1, small T antigen and large T antigen RT-PCR products from patient RNA.

Histology confirmed the diagnosis of MCC for recurrences (Fig. 2A–C) and MCCs were confirmed to be Merkel cell polyoma virus (MCPyV) positive by reverse transcriptase-polymerase chain reaction (RT-PCR) of tumor RNA (Fig. 2D), which detected RT-PCR products for MCPyV VP1, small T antigen and large T antigens, using the MCPyV VP1-specific primer set: forward 5’CAACGAAAATTTGCCAGCTTA-3′ and reverse 5’TTTAACAGAATATTGCCTCCCAC-3′; the MCPyV small T antigen-specific primer set: forward 5’-TGCCACCAGTCAAAACTTTC-3′ and reverse 5’-AGCAAAAAAACTGTCTGACGTG-3′, and the MCPyV large T antigen-specific primer set 5’-AAGGACCCATACCCAGAGGAAG-3′ and reverse 5’−CCAACTCAAGATCCAGAAAGCC-3′, in 35 cycle RT-PCR reactions of 1μl undiluted reverse transcription reaction from 0.5μg of purified tumour RNA, and annealing temperatures of 54 °C for VP1 and small T antigen primers and 56 °C for large T antigen primers.

**Fig. 3:**
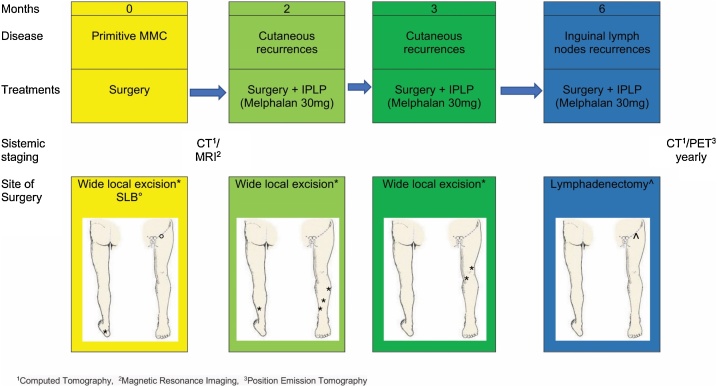
Timeline of treatment.

One month following initial treatment, the patient developed subcutaneous recurrences in the thigh of the same limb and was subjected to a further round of surgery combined with Melphalan IPLP. Within 3 months, the patient also developed an inguinal lymph node recurrence and was subjected to a third round of surgery combined with Melphalan IPLP (Fig. 3). Fig. 3 illustrates the timeline of treatment and location of cutaneous recurrences.

The IPLP technique employed in this particular case was identical to that previously reported in detail and employed for the treatment of melanoma [9,10]. Perfusion was conducted in normothermic or mild hyperthermic conditions, using a single dedicated and integrated circulation device (Performer LRT; RanD, Medolla, Italy), in order to maintain hyperthermia in the extracorporeal circuit and ensure normothermia or mild hyperthermia in the patient and at the end of perfusion, systemic Melphalan toxicity was reduced by chemo-filtration.

Local toxicity was not observed following treatments and supportive therapy with granulocyte colony-stimulating factor restored the grade 3 neutropenia (Common Terminology Criteria for Adverse Events of the National Cancer Institute (CTCAE v4.03)), detected after each procedure, back to normal within approximately two weeks. Patient follow-up after the last treatment, consisted of clinical examination with CT scan every six months and a yearly PET scan, in line with National Comprehensive Cancer Network (NCCN) guidelines. At 56 months after the last IPLP this patient is still alive and in complete remission with no evidence of disease.

## 3 Discussion

MCCs exhibit a high potential for local recurrence and systemic dissemination with surgery, with or without radiotherapy, used to manage locoregional disease and minimize local recurrence. Recommendations, according to the clinical and/or microscopic lymph node status, include wide surgical resection, sentinel lymph node biopsy and complete lymph node dissection with radiotherapy of the nodal basin. This approach, however, associates with a 26–60% locoregional recurrence rate within 2 years [11], either as true recurrences or in transit metastases (ITMs), with lymphatic ITMs developing anywhere between the primary tumor and draining lymph node basin. MCC patients with locoregional recurrence exhibit a 3-year survival-rate of 39% [11], and patients with recurrent ITMs exhibit a 3-fold increased risk of subsequent locoregional recurrence compared with initial local or nodal locoregional recurrence. Within this context, therapeutic options are limited and challenging and associate with disease states that range from a few microscopic to bulky lesions in previously treated extremities.

Radiotherapy, represents a palliative option for cutaneous metastases but does not appear to alter the course of stage IIIc disease nor improve overall survival, principally due to the fact that ITMs are often multifocal and tend to localize to the curved surfaces of extremities adjacent to previously treated fields, adding to the technical difficulty of radiotherapy [12]. Systemic chemotherapeutic regimens are mainly reserved for patients with metastatic disease or as palliative therapy for disabling symptoms [13,14]. In contrast, novel immune checkpoint inhibitors have greatly improved outcomes but are reserved for patients with metastatic MCC and not recommended for patients with chronic infections, including Hepatitis C [1].

Clinical outcomes for MCC patients with limb ITMs could be improved by techniques that deliver high drug concentrations to the affected region, with potential for repetitive application, limited systemic toxicity and acceptable morbidity. In melanoma patients with ITMs, locoregional chemotherapy has been performed using the following techniques: i) Isolated Limb Infusion-ILI [15], ii) Isolated Limb Perfusion-ILP [16], and iii) isolated Pelvic and Limb Perfusion-IPLP with chemo-filtration, as consequence of pelvic and/or inguinal location [9], procedure that is interchangeable and practicable with both surgical or percutaneous approaches [17,18]. The similarity between cutaneous melanomas and MCCs has prompted the use of similar therapeutic procedures to treat MCC with locoregional recurrences and/or ITMs, with a recent study reporting a high rate of complete response with long-term control of locoregional recurrence, using ILP with Melphalan in stage IIIb and IIIc MCCs [5,7]. Complete responses have also been reported for MCC patients with ITMs, subjected to either ILI or ILP [19,6].

In this article, we report the case of a patient presenting with cirrhosis related to active chronic hepatitis C infection and multiple cutaneous MCC recurrences in the left inferior limb, who responded successfully to multiple treatments, consisting of surgery combined with locoregional IPLP chemotherapy, that highlights several points of interest. First, the patient was not eligible for innovative systemic immune checkpoint inhibitor therapeutic clinical trials due to active chronic Hepatitis C-infection and treatment with NS5B inhibitors. Second, the patient was also not suitable for palliative radiotherapy or standard systemic chemotherapy due to severe chronic neutropenia. Consequently, we opted for IPLP therapy, followed immediately by chemo-filtration in order to reduce the severity of systemic toxicity. Third, the peculiarity of this case is the repetition of surgical excision and IPLP perfusion. In our previous reports, positive results in advanced melanomas were obtained following the first and second therapeutic cycles and also following several additional cycles, as in other advanced cancer patients [20], with survival rates significantly increased when more than two cycles were performed [10,18]. Finally, employing IPLP, we were able to treat both the affected limb and pelvic region, whereas employing ILI or ILP it is only possible to infuse or perfuse the median and distal part of the thigh together with the leg. In aggressive tumors, such as MCCs, high chemotherapeutic drug concentrations may facilitate better control of subclinical groin and pelvic relapse-sites.

## 4 Conclusion

In conclusion, surgical excision combined with hypoxic Melphalan IPLP perfusion, despite its application as a palliative therapy in an MCC patient with locoregional recurrences, resulted in unexpected long-term survival. This suggests that MCC patients that are non-responsive or not eligible for systemic therapies, should be assessed in a clinical trial to further investigate the potential efficacy of locoregional chemotherapy in this aggressive tumor type.

## Funding

There are no sources of funding.

## Ethical approval

This retrospective case report study has been approved by the investigational review board [Ethics committee of “ASL n.1, Abruzzo, Italy; Chairperson: G. Piccioli; protocol number 10/CE/2018; date of approval: 19 July 2018 (n.1419)].

## Consent

This study on patient possesses ethics committee approval.

Patient expressed a written informed consent to accept the publication of this paper.

## Author contribution

Author statement about individual contributions: Guadagni S: Conceptualization; Clementi M: Conceptualization; Puccica I: Data curation; Valiyeva S: Data curation; Chiominto A: Methodology; Mackay AR: Methodology: Farina R: Methodology; Cappabianca L: Methodology; Guadagni S: Validation; Clementi M: Writing - original draft; Guadagni S: Writing - original draft; Mackay AR: Writing - review &editing.

Authorship: All authors provided substantial contributions to the following: (1) the conception and design of the study; (2) drafting the article or revising it critically for important intellectual content; (3) final approval of the version to be submitted.

## Registration of research studies

Rsearchregistry 4805.

## Guarantor

Prof. Guadagni Stefano.

## Provenance and peer review

Not commissioned, externally peer-reviewed.

## Declaration of Competing Interest

None.
